# Platelet autologous growth factors decrease the osteochondral regeneration capability of a collagen-hydroxyapatite scaffold in a sheep model

**DOI:** 10.1186/1471-2474-11-220

**Published:** 2010-09-27

**Authors:** Elizaveta Kon, Giuseppe Filardo, Marco Delcogliano, Milena Fini, Francesca Salamanna, Gianluca Giavaresi, Ivan Martin, Maurilio Marcacci

**Affiliations:** 1 Biomechanics Laboratory-Rizzoli Orthopaedic Institute, Bologna, Italy; 2 Laboratory of Preclinical and Surgical Studies-Rizzoli Orthopaedic Institute, Bologna, Italy; 3 University Hospital Basel, Basel, Switzerland

## Abstract

**Background:**

Current research aims to develop innovative approaches to improve chondral and osteochondral regeneration. The objective of this study was to investigate the regenerative potential of platelet-rich plasma (PRP) to enhance the repair process of a collagen-hydroxyapatite scaffold in osteochondral defects in a sheep model.

**Methods:**

PRP was added to a new, multi-layer gradient, nanocomposite scaffold that was obtained by nucleating collagen fibrils with hydroxyapatite nanoparticles. Twenty-four osteochondral lesions were created in sheep femoral condyles. The animals were randomised to three treatment groups: scaffold, scaffold loaded with autologous PRP, and empty defect (control). The animals were sacrificed and evaluated six months after surgery.

**Results:**

Gross evaluation and histology of the specimens showed good integration of the chondral surface in both treatment groups. Significantly better bone regeneration and cartilage surface reconstruction were observed in the group treated with the scaffold alone. Incomplete bone regeneration and irregular cartilage surface integration were observed in the group treated with the scaffold where PRP was added. In the control group, no bone and cartilage defect healing occurred; defects were filled with fibrous tissue. Quantitative macroscopic and histological score evaluations confirmed the qualitative trends observed.

**Conclusions:**

The hydroxyapatite-collagen scaffold enhanced osteochondral lesion repair, but the combination with platelet growth factors did not have an additive effect; on the contrary, PRP administration had a negative effect on the results obtained by disturbing the regenerative process. In the scaffold + PRP group, highly amorphous cartilaginous repair tissue and poorly spatially organised underlying bone tissue were found.

## Background

The incidence of articular cartilage lesions has grown due to the marked increase in sports participation and greater emphasis on physical activity in all age groups; patient expectations about recovery have also risen. Unfortunately, articular chondral defects, with their inherent limited healing potential, are hard to treat and remain a challenging problem for orthopaedic surgeons. Promising results are obtained with the tissue engineering approach, and matrix-assisted autologous chondrocyte transplantation is now widely used in Europe [1,2]. However, the results obtained for the treatment of cartilage lesions are still controversial, and the treatment of osteo-cartilaginous lesions is even more problematic because tissue damage also extends to the sub-chondral bone, involving two different tissues characterised by different intrinsic healing capacities. Several authors have highlighted the need for biphasic scaffolds to reproduce different biological and functional requirements for guiding the growth of the two tissues, especially in large osteochondral defect repairs [3,4].

Following this rationale, in a previous animal study we tested a recently developed composite scaffold, composed of Type I collagen and nanostructured hydroxyapatite (HA), which mimics the biochemical and biophysical properties of the different layers of native osteochondral structures [5,6]. This study highlighted the promising potential of the graded, biomimetic osteochondral scaffold in promoting bone and cartilage tissue restoration without necessarily including autologous cells [6]. In the present study, we tested if the combination of scaffold and autologous platelet-rich plasma (PRP) would further improve the good results previously obtained with the scaffold alone by increasing the regeneration process of the osteochondral unit. The addition of growth factors (GFs) might enhance the results and improve scaffold integration and tissue replacement. PRP is a method that enables to obtain in a simple, inexpensive and minimally invasive way many autologous growth factors and actually is widely experimented in different fields of medicine for its potential in aiding tissue regeneration. The rationale for the use of PRP in the treatment of many different tissues is that it provides a reservoir of critical GFs and cytokines, which govern and regulate the tissue healing process and is quite similar in all kinds of tissues. In fact, many in vitro studies suggested that growth factors contained in PRP may be useful for enhancing both the chondral and osseous component of the osteochondral regeneration induced by the scaffold. Platelet concentrates play an important role in the bone tissue reparative processes by stimulating the initial recruitment of bone marrow cells for migration [7], mitogenesis, differentiation into osteoblasts, and angiogenesis [8]. It has also been shown that the addition of PRP enhances chondral regeneration, enhancing the proliferation of chondrocytes and improving the biosynthesis of the cartilage matrix proteins [9,10]. Regarding in vivo studies, so far, few studies have been performed on PRP for osteochondral regeneration compared with the extensive work on bone and other tissue healing [11-13].

The purpose of this experimental study was to evaluate the potential of PRP to enhance the osteochondral regeneration process induced by the newly developed collagen-hydroxyapatite scaffold in a sheep model.

## Methods

### Study design

In the present study, we experimented with two different kinds of tissue repair strategies: a) biomimetic scaffold alone (S-Group) and b) biomimetic scaffold combined with PRP (S-PRP Group). Spontaneous cartilage and bone repair was used as control group (C-Group).

Animal care and surgery were approved by the technical scientific and ethical committees of our institute and were performed under national and European regulations (Law by Decree n. 116/92).

Twelve skeletally mature female adult sheep (Bergamasca-Massese, 70 ± 5 kg b.w.) were acquired from authorised farms and quarantined for at least 7 days before use. The animals were randomly divided into 3 groups of 4 animals each according to the treatment group: S-Group, S-PRP Group and C-Group. A total of 24 osteochondral lesions were performed on the right medial and lateral femoral condyles, and each animal received the same treatment on both condyles. Animal-free movement was permitted immediately after surgery. After two weeks, the sheep were returned to the external stabling fields until the end of the experimental time (6 months).

### Scaffold preparation

**Figure 1:**
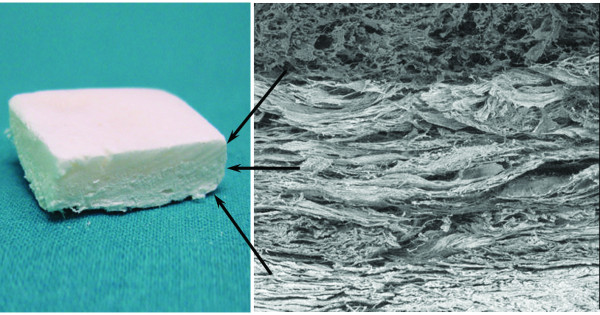
**Three-gradient, multi-layer scaffold that mimics the articular osteochondral compartment, reproducing both molecularly and morphologically cartilage and the subchondral bone layer**.

The osteochondral (OC) biomimetic scaffold (Fin-Ceramica Faenza S.p.A., Faenza-Italy) has a porous, 3-D, composite, tri-layered structure, mimicking the whole osteochondral anatomy. The cartilaginous layer, consisting of Type I collagen, has a smooth surface to maintain joint congruency. The intermediate layer (tidemark-like) consists of a combination of Type I collagen (60%) and HA (40%) whereas the lower layer consists of a mineralised blend of Type I collagen (30%) and HA (70%), reproducing the subchondral bone layer (Figure 1).

Each layer of the scaffold is separately synthesised by a standardised process, starting from an atelocollagen aqueous solution (1% w/w) in acetic acid, isolated from equine tendon; the upper non-mineralised chondral layer of the scaffold is obtained by dissolving 200 g of acetic solution of Type I collagen (Opocrin S.p.A., Corlo di Formigine, Modena, Italy) in 200 ml of bi-distilled water after setting the pH at 5.5. By adding 0.1 NaOH, the precipitate is homogenised by moderate stirring and rinsed in distilled water. The assembled collagen fibres are subsequently cross-linked with 42 ml of 0.5 g/L 1,4-butanediol diglycidyl ether (BDDGE) solution (Fluka, Sigma-Aldrich Group) and stored at 4°C for 48 hours.

The intermediate and lower layers of the scaffold are obtained by nucleating bone-like, nanostructured, non-stoichiometric hydroxyapatite into self-assembling collagen fibres, as occurs in the natural biological neo-ossification process [14]. The mineralised intermediate layer is obtained starting from two reagents prepared as follows: reagent A, prepared by diluting 300 g of Type I collagen acetic solution with H_3_PO_4 _40 mM, to reach a final pH of 3.0; reagent B, prepared by mixing 480 ml of a 42 mM Ca(OH)_2 _solution with 20 ml of 48 mM MgCl_2_·6H_2_O solution and 24 ml of SBF (Simulated Body Fluid). Under gentle stirring conditions, reagent A is dripped into reagent B until hydroxyapatite nanoparticles are nucleated into the auto-assembled collagen fibres, reaching a final pH of 6.0. The obtained precipitate, composed of 60% collagen and 40% of hydroxyapatite, is rinsed in distilled water, cross-linked with 63 mL of BDDGE cross-linking solution, and stored at 4°C for 48 hours.

The lower layer of the scaffold is also prepared starting from two reagents: reagent C, obtained by adding 40 mM H_3_PO_4 _to 200 g of Type I collagen acetic solution_, _achieving a pH of 3.0; reagent D, obtained by mixing 1100 ml of 42 mM Ca(OH)_2 _solution with 50 ml of 48 mM MgCl_2_·6H_2_O solution and 55 ml of SBF (Simulated Body Fluid). Under stirring conditions, reagent C is dripped into reagent D until the precipitation of HA into auto-assembled collagen fibres with a final pH of close to 7.0. The composite precipitate is 70% HA and 30% collagen, respectively. Subsequently, after thoroughly rinsing in bi-distilled water, self-assembled collagen HA fibres are cross-linked with 63 mL of BDDGE solution and then stored at 4° C for 48 hours.

The final construct is obtained by physically combining the upper, intermediate and lower layers on top of a Mylar sheet. The three layers are piled up; then, a knitting procedure is applied at each interface (bone-tidemark interface and tidemark-cartilage interface) to assure good integration by the exchange of anchor fibres between the layers and to avoid delamination at the interface. Finally, they are freeze-dried and gamma-sterilised at 25 KGray [14].

### PRP preparation

PRP was prepared according to the method described in 2004 by Weibrich et al. [15] as follows: before each operation (within about 1 h), approximately 20 ml of peripheral venous blood was drawn from the radial vein into siliconised tubes containing 3.8% sodium citrate at a blood/citrate ratio of 9/1. PRP was obtained by centrifugation at 1000 r.p.m. for 5 min.

The number of platelets (PLTs) was determined on whole blood and on PRP under a microscope with a haemocytometer after 1/100 dilution with ammonium oxalate (Unopette1, Becton Dickinson, Milan, Italy). The % yield was calculated in the following way: (number of PLTs in PRP/number of PLTs in the whole blood)x100. The mean ± sd of PLTs number in whole blood was 281 ± 56 × 10^3^/μl; after centrifugation the mean number of PLTs in the PRP was 874 ± 87 × 10^3^/μl with a mean % yield of 316 ± 36%.

For PLT activation, a 10% solution of CaCl_2 _(Sigma) in a 50 μl/ml proportion was added to PRP immediately before use. Two millilitres of PRP were soaked into the scaffold.

Aliquots of plasma and PRP were dispensed in Eppendorf tubes for storage at -70°C. Plasma, PRP and PRP after activation with CaCl_2 _were assayed for Transforming Growth Factor β1 (TGF-β1, ELISA immunoassay, R&D Systems, MN, USA), Platelet Derived Growth Factor AB (PDGF-AB, ELISA immunoassay, R&D Systems, MN, USA), and Interleukin 1 (IL-1, ELISA immunoassay, R&D Systems, MN, USA).

### Surgical procedure

Surgery was conducted under general anaesthesia following a standardised protocol: pre-medication with 10 mg/kg ketamine i.m. (Ketavet 100, Farmaceutici Gellini S.p.A., Aprilia, Latina, Italy), 0.3 mg/kg xylazine i.m. (Rompun Bayer AG, Leverkusen, Germany) and 0.0125 mg/kg atropine sulphate s.c.; induction with 6 mg/kg sodium thiopentone i.v. (2.5%); and maintenance with O_2_, NO_2 _and 2-3% fluothane (Halothan, Hoechst AG, Germany) under assisted ventilation (Servo Ventilator 900 D, Siemens, Germany). After medial arthrotomy, a parapatellar approach to the lateral and medial condyles was performed, and the condyle was exposed with the animals in dorsal recumbence and the surgery limb in a supine and maximally bent position. Then, an osteochondral lesion was made on the medial and lateral femoral condyles. A specifically designed drill was used to create a 7-mm diameter and 9-mm thick defect in the weight-bearing area of both condyles. Grafts were implanted following the press-fit technique. A total of sixteen osteochondral grafts were implanted: 8 osteochondral grafts were soaked with PRP at least 5 min before implantation. Eight lesions were left untreated and used as controls.

Antibiotics (cephalosporin, 1 g/day for 5 days) and analgesics (ketoprofen 500 mg/day for 3 days) were administrated post-operatively, and a veterinarian evaluated the animals' welfare.

### Explanting of samples

All animals were sacrificed 6 months after surgery under general anaesthesia and by injection of Tanax^® ^(Intervet-Italia S.r.l., Peschiera Borromeo, Milan, Italy). The stifle joints were carefully opened; the patellar ligaments were dorsally reflected; and the joint capsule was macroscopically evaluated for signs of inflammation, such as tissue reddening, hypertrophy of the villous part of the synovial membrane, tissue adhesions, appearance of a fat pad, and clearness and colour of the synovial fluid. Both condyles of the joint were carefully exposed, thus preserving the surface of the operation in the medial and lateral areas. The newly formed tissue in the areas of scaffold implantation was macroscopically assessed. The cartilage surface was evaluated for position, surface and staining of the implanted areas and the implant-host cartilage interface. Obvious signs of matrix degradation, such as fibrillation, cleft formation, cobble stone appearance, and discoloration of the hyaline cartilage surface were recorded and compared among groups. Each explanted knee was cut into medial and lateral condyles. Subsequently, the areas of the operation were carefully removed, paying attention also when removing portions of healthy cartilage surrounding the lesion. The macroscopic appearance of the implants and the quality of healing were blindly assessed using a modified scoring system from Fortier [16] that analyses surface texture of repair tissue, percent area of the defect filled, and graft-recipient tissue integration. The score was modified to add the bone-cartilage appearance and bone-defect filling evaluation. We also made a gross morphological evaluation using a scoring system proposed by Niederauer [17], focusing on edge integration, smoothness of the cartilage surface, cartilage degree of filling, and colour of the cartilage.

### Sample preparation and histological evaluation

Each medial and lateral sample was cut in half along the central axis of the implant. Half remained undecalcified and were processed for resin embedding; half were decalcified and embedded in paraffin. The latter samples were fixed in 10% formalin-buffered solution for 72 hours and decalcified in a nitric/formic acid solution. When decalcification was complete, the samples were dehydrated in a graded series of alcohols from 70% to absolute and then processed for paraffin embedding. Four-micrometer-thick sections were obtained by a Microm HM340E (Microm International GmbH, Heidelberg, Germany) and stained with toluidine blue, fast green and haematoxilin/eosin or safranin-O, fast green and toluidine blue.

The specimens of undecalcified bone processing histology were first fixed in 4% buffered paraformaldehyde for 48 hours, then dehydrated in graded series of alcohols until the absolute was reached. Finally, they were embedded in an epoxy resin (Struers Co. Copenhagen, Denmark). Blocks were sectioned along a plane parallel to the long axis of the osteochondral transplant. A series of 200 ± 10-μm thick sections, spaced 300 μm apart (due to a microtome diamond saw thickness), was obtained with a Leica 1600 diamond saw microtome (Leica SpA, Milan, Italy). Then, sections were thinned to 30 ± 10 μm and stained with safranin-O/fast green and acid fuchsin. Microradiography evaluations were performed on 100 ± 10-μm sections, and images were acquired by high resolution photo-emulsion plates (exposition: 30 seconds, 20 kV).

The sections were processed for routine histological analysis by using a transmission and polarised light Axioskop Microscope (Carl Zeiss GmbH, Jena, Germany) and a computerised image analysis system with Kontron KS 300 software (Kontron Electronic GmbH, Eiching bei Munchen, Germany). After light microscopy evaluation at different magnifications, bone measurements were taken semi-automatically by 2 blinded investigators at a magnification of 10×. The histology was assessed using the score proposed by Niederauer [17].

### Immunohistochemical analysis

The sections of decalcified and paraffin-embedded samples were deparaffinised, incubated with trypsin 0.1% for 20 min at 37°C, and then incubated with goat serum diluted 1:20 for 10 min. The specimens were then incubated for 1 hour at room temperature with a primary monoclonal antibody against type II collagen (clone II-II6B3; Developmental Studies Hybridoma Bank, Baltimore, MD) or for 24 hours at 4°C with a primary monoclonal antibody against type I collagen (Quartett, Berlin, Germany). The slides were then incubated with a biotinylated goat anti-mouse secondary antibody (Dako, Carpenteria, CA) for 35 min. Finally, the avidin-biotin complex coupled with alkaline phosphatase (ABC/AP complex, Dako, Carpenteria, CA) was used for the staining according to the manufacturer's instructions followed by incubation with fast red substrate and levamisole (Dako, Carpenteria, CA) and counterstaining with haematoxylin/eosin.

### Statistical analysis

Statistical analysis was performed by SPSS 15.0. Data were expressed in terms of mean ± SD at a significance level of *p *< 0.05. The non-parametric Kruskal-Wallis test or Mann-Whitney tests, followed by the Monte Carlo methods to compute probability, were used to analyse data.

## Results

### Platelet growth factors

The results of TGF-β1 (activated fraction), PDGF-AB and IL-1 was 53 ± 7, 62 ± 14, 1.6 ± 0.4 ng/ml in plasma, 113 ± 28, 87 ± 11, 3.3 ± 0.4 ng/ml in PRP and 453 ± 110, 232 ± 85, 9.7 ± 5 ng/ml in PRP activated with CaCl_2_, respectively.

### Gross appearance and macroscopic evaluation

All animals tolerated surgery well and survived the post-surgical period. Gait was completely normal without a severe limp, and the joints appeared stable. All sheep showed soft tissue swelling of the treated joints. There were no signs of synovitis. No signs of fracture were detected on post-operative and pre-operative X-Ray examination.

The joint inspection revealed the absence of joint inflammation, adhesions between the joint capsule, fat pad and collateral joint compartment. A slightly hyperemic synovium was observed in the majority of cases. No synovial hypertrophy or fibrosis was noted. All grafts were still in their original location. Small osteophytes were detected in the medial condyles of all groups.

**Figure 2:**
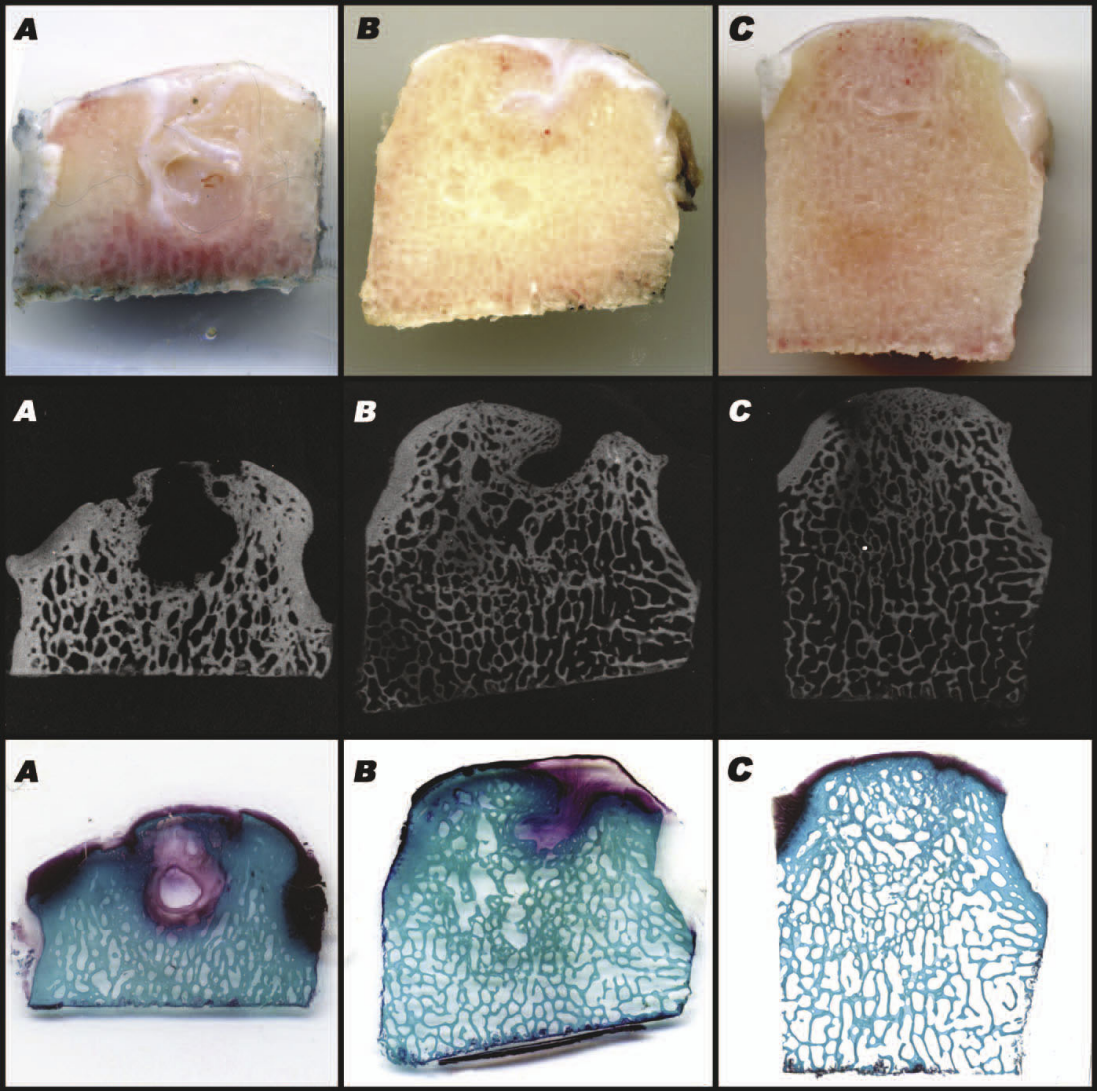
**Macroscopic, micro X-ray and histological evaluations at 6 months of the C-Group (A), S-PRP Group (B), S-Group (C)**.

Gross evaluation of specimens showed that no bone and cartilage defect healing occurred in the control group (Figure 2A). Good integration of the healthy chondral surface, newly formed hyaline-like tissue and better bone regeneration were observed in the group implanted with the scaffold alone (S-Group) (Figure 2C) whereas incomplete bone defect filling and irregularity of the bone-cartilage surface were detected in the S-PRP Group (Figure 2B).

**Figure 3:**
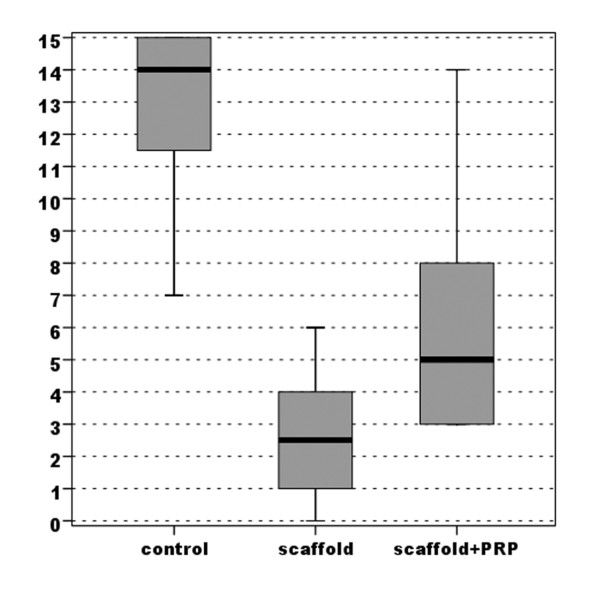
**Comparison of scores obtained with the modified Fortier evaluation of gross appearance (max 0-min 15)**.

**Figure 4:**
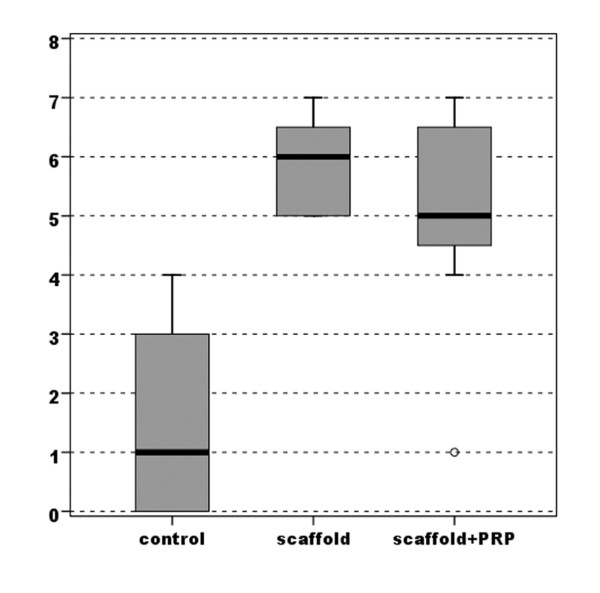
**Comparison of scores obtained with the Niederauer evaluation of gross appearance (max 8-min 0)**.

Evaluation of the macroscopic appearance with the modified Fortier [16] and Niederaurer [17] scores showed better results in both experimental groups compared with the control group. However, the S-Group achieved marked improvement with a highly statistically significant difference (*p *< 0.0005), whereas the group with PRP added to the scaffold obtained lower improvement with a lower statistically significant difference compared with the untreated group (*p *= 0.018 for the Niederauer score and *p*= 0.024 for the modified Fortier score) (Figures 3, 4) (Table 1).

**Table 1:** Macroscopic appearance of the implants and the quality of healing assessed using a modified scoring system from Fortier and a scoring system proposed by Niederauer.

Fortier score (max 0-min 15)			Niederauer score (max 8-min 0)		
**C-Group**	**S-PRP Group**	**S-Group**	**C-Group**	**S-PRP Group**	**S-Group**

11	7	6	3	5	6

12	3	4	3	6	5

15	5	1	0	5	7

15	14	1	0	1	5

14	5	3	2	5	6

7	3	2	4	7	5

15	9	4	0	4	6

14	3	0	0	7	7

**12.86**	**6.13**	**2.63**	**1.50**	**5.00**	**5.88**

### Microradiographic evaluation

Microradiographic evaluation highlighted the appearance of newly formed bone in the deepest area of the osteochondral implant (Figure 2): an improvement in subchondral bone healing was observed in both experimental groups compared with the control. Better bone regeneration in the S-Group compared with the S-PRP Group was confirmed (Figure 2). No bone growth in the chondral layer was observed in any group.

### Histological evaluation

**Figure 5:**
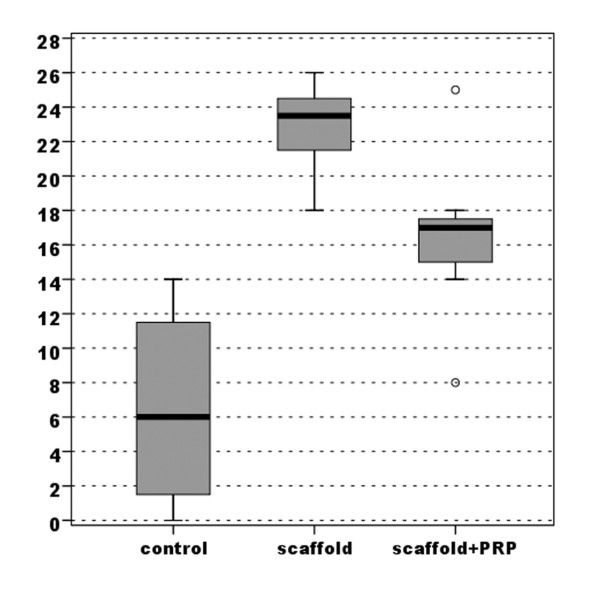
**Comparison of scores obtained with the Niederauer histologic scoring scale for osteochondral defects (max 28-min 0)**.

**Figure 6:**
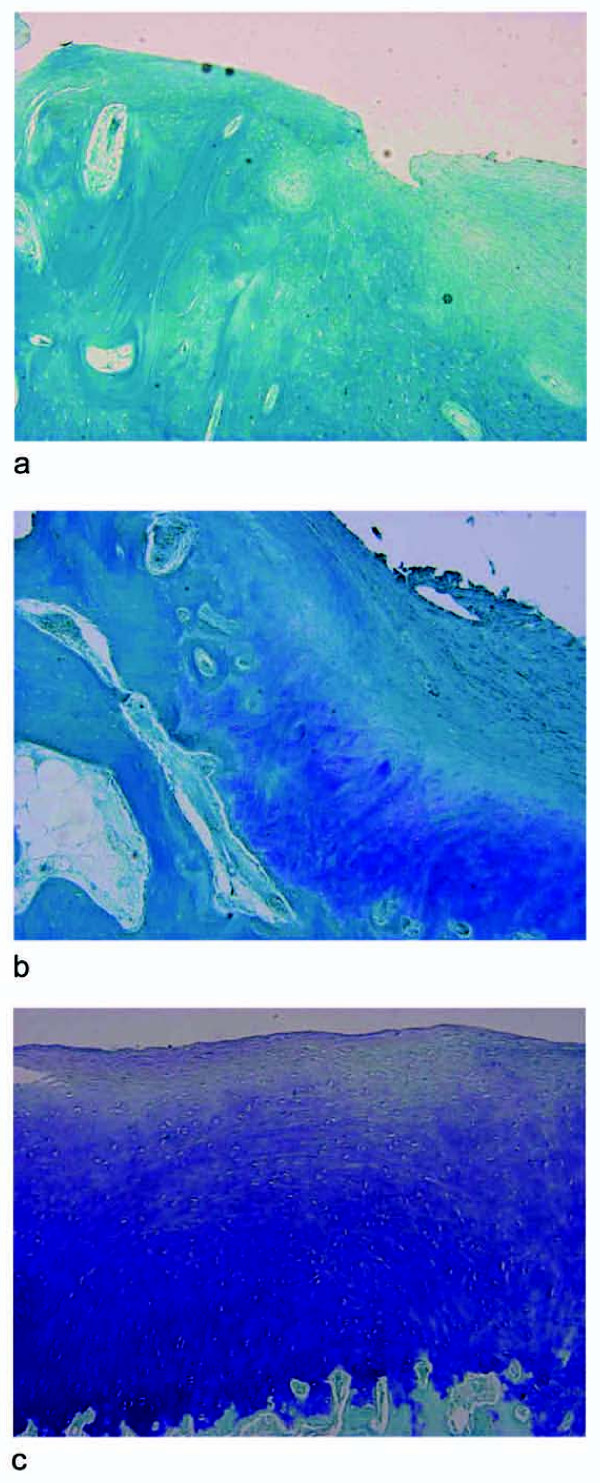
**Histological evaluation at 6 months of undecalcified samples of the C-Group (A), S-PRP Group (B), S-Group (C)**. Staining with fuchsin acid/fast green/toluidine blue, 10× magnification.

Histological assessment confirmed the macroscopic results (Figure 2). No bone and cartilage defect healing occurred in the control group; the defect was filled with amorphous fibrous tissue. Both experimental groups displayed significantly superior histological results compared with the control group. Comparing the experimental groups, worse results were observed in the S-PRP Group (*p *= 0.036) (Figures 5, 6). Neither an inflammatory reaction nor giant cells were observed in the grafted area, and complete reabsorption of the implanted biomaterial was detected. Histological evaluation showed the presence of newly formed repair tissue and good integration of the scaffolds with the host cartilage for the S-Group. The presence of a newly formed hyaline-like tissue with strong proteoglycan staining and the columnar rearrangement of chondrocytes were observed (Figure 6). An underlying, well-structured, subchondral trabecular bone, distinguishable from the healthy adjacent bone, was also noted. Also better bone regeneration was observed in the S-Group compared with the S-PRP group where incomplete bone defect filling and irregularity of the bone-cartilage surface were detected.

**Figure 7:**
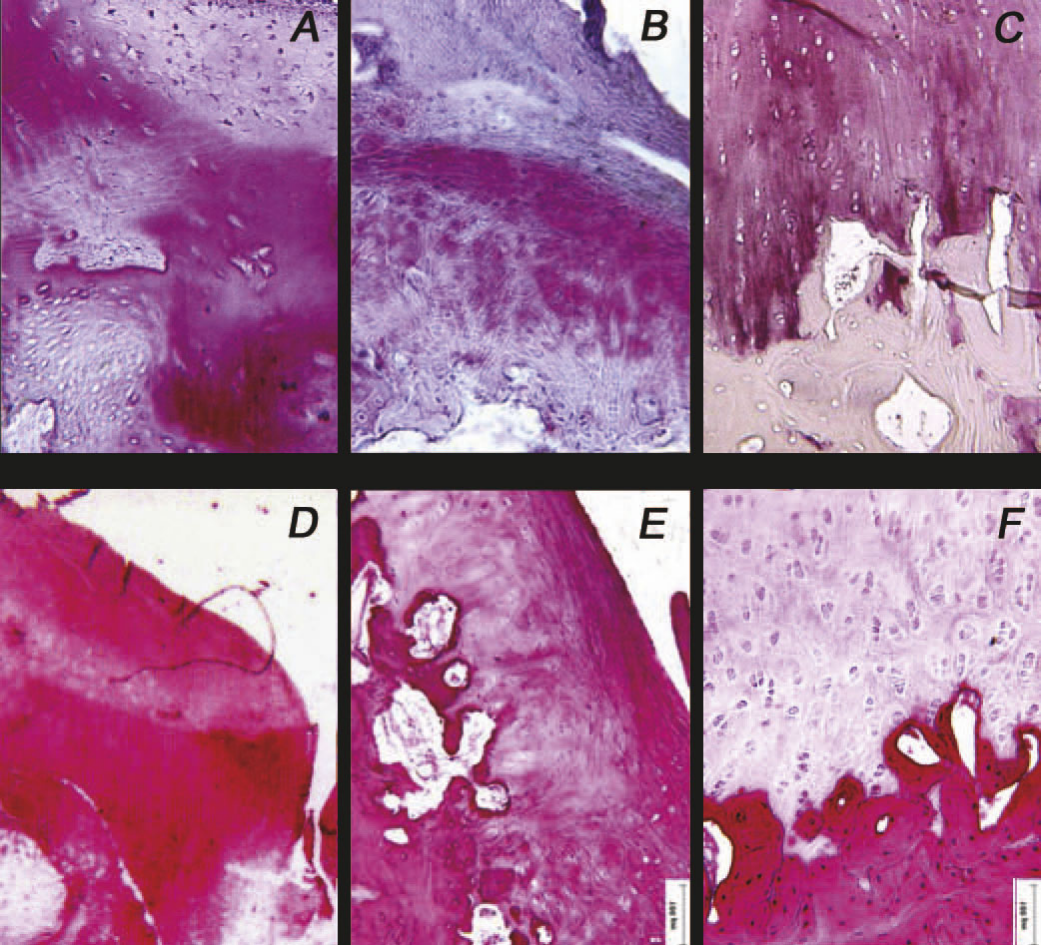
**Representative fields of immunohistochemical staining for type II (A, B, C) and type I (D, E, F) collagen of decalcified samples of the C-Group (A, D), S-PRP Group (B, E) and S-Group (C, F) at 6 months**. Bar = 100 microns.

Immunohistochemical staining for collagen types I and II generally confirmed the histological evaluation. The S-Group displayed an orderly pattern of tissue repair with type II collagen staining positive in the cartilaginous layer down to the interface with the subchondral bone, and type I collagen staining uniformly positive in the subchondral tissue or associated with single cells in the chondral region (Figure 7 C, F). The subchondral tissue included discrete areas that were positive for type II collagen; the hypertrophic chondrocyte morphology suggested ongoing remodelling towards a bone matrix. Instead, in the S-PRP and C-Groups, type II collagen staining was either negative or positive in scattered areas whereas a positive staining for type I collagen in the extracellular matrix was extended throughout the repair tissue up to the joint space (Figure 7 A, B, D, E).

## Discussion

The aim of this study was to evaluate the potential of autologous PRP to enhance the osteochondral regeneration process induced by a newly developed biphasic scaffold in a sheep model.

The sheep model was chosen for the current experiment because limb-loading in the sheep is comparable to that of humans and more similar than the rabbit model to the human one. Even if many questions on tissue regeneration can be answered in relatively simple models, in small animals, the level of the load cannot be a critical factor for the success of a tissue-engineered procedure. It is generally agreed that the results obtained in large animals are more representative of the clinical situation. Final pre-clinical tests in large animals present relevant loading conditions and allow the adoption of a surgical technique similar to the final procedure used in humans [13,18].

Both lateral and medial condyle defects underwent the same treatments to avoid the possible influence on defect healing among the investigated treatments. In addition, as reported by other authors, both defects were created on the same knee to allow the sheep to unload the operated limbs for the first post-operative week [19]. In agreement with other researchers and our previous experiences, no difference in regeneration between the medial and lateral condyles was observed although it is known that the medial compartment of the knee sustains higher loads than the lateral one [20]. As also shown in a previous study [6] with the same animal model, osteochondral defects treated with a scaffold alone showed the appearance of a newly formed cartilaginous mantle similar but not equal to the native hyaline cartilage. We must keep in mind that the kinetics of cartilaginous and bone regrowth certainly exceed a 6-month follow-up; therefore, the processes of connective tissue repair were probably still in evolution at the time of evaluation. However, the 6-month follow-up was evidently sufficient to provide clear indications of the ongoing regenerative processes. Whereas the results indicated poor spontaneous healing of the osteochondral defect in this sheep model, the implant of the gradient biomimetic scaffold led to the reconstruction of the hyaline-like cartilage and the structured bone tissue anchored to the interface of adjacent healthy tissues. Healing of the deep osteochondral defect was evident: histological images excluded the presence of bone tissue inside the upper cartilaginous layer and, from a histological viewpoint, no inflammatory reactions were observed at any of the treatment sites. Moreover, osteochondral defects treated with the biphasic scaffold had the appearance of a newly formed cartilaginous mantle similar to the native hyaline cartilage.

Although the marked regrowth observed of good quality subchondral bone characterised by well-defined cortex, in some other areas osteoid tissue appeared to be still distinguishable, and also the cartilage regenerative process was partial with the presence of only hyaline-like cartilage. To further improve the osteochondral regeneration capacity of this 3-layered, biomimetic, nanostructured scaffold, the addition of bioactive agents was experimented aiming to speed the reparative process and led to a higher tissue quality. PRP is a recently developed, promising method that provides an easy, safe and cost-effective way to obtain various GFs, which have been shown in vitro to increase chondrocyte proliferation and differentiation markers [9,10]. Wu et al. also hypothesised that the multiple GFs contained in PRP could have a synergistic effect on osteoblasts, which should improve proliferation and synthesis of bone marrow precursors and improve the integration of regenerated cartilage and underlying bone tissue [11].

Surprisingly, despite the in vitro premises and the positive theoretical assumptions, the results of this study showed not only the lack of a positive effect but even a negative influence of autologous PRP on bone and cartilage regeneration. Findings of our research were a highly amorphous cartilaginous repair tissue and a poorly spatially organised underlying bone tissue.

Analysing the literature on chondral and osteochondral tissue regeneration, few studies have been performed until now although there have been several papers about bone healing. In a rabbit model, Sun et al. demonstrated that the addition of PRP to a scaffold made of polyglycolic acid had a significantly positive effect on osteochondral formation [12]. In a goat model, Brehm et al. evaluated the repair of osteochondral defects with an autologous scaffold-free cartilage construct alone and with the addition of PRP as an adhesive to secure the implant or to cover the overlying periosteal flap [13]. Even if their study was aimed at evaluating different implantation modalities, they found that PRP did not improve integration of the implant. Moreover, when PRP was used in combination with a periosteal flap, it was less effective than the periosteal flap alone. Finally, PRP alone also caused intensive bone remodelling of the subchondral bone [13].

These heterogeneous findings are certainly difficult to explain, but in the most investigated field of bone healing, discordant results have been obtained when PRP was added to various biological and synthetic biomaterials. No advantages on the combination of hydroxyapatite, beta-tricalcium phosphate and PRP were reported in experimental studies in the early phase of bone healing [21]. Some authors report the lack of beneficial effects when PRP was added to endosseous implants both in cortical and trabecular bone [15,22,23]. Mechanical trauma due to high pressure after gel application in press-fit implant surgery has been hypothesised [23] as a negative factor that may compromise the final outcome. Better results have been obtained from other authors using PRP, but the effects depended on the implant chemical surface, being more effective when applied on metallic surfaces, whereas to a lesser extent on ceramic surfaces [23-27].

Analysis of the results obtained in the different experiments reported in the literature is complicated. In fact, when discussing PRP, several variables have to be considered. Although our interest was not to afford the biological variables linked to PRP therapy, the following considerations are highlighted. These variables mainly depend on the methods of PRP preparation and conservation, the type of activators, the type of pathology treated, the dose used, and the mode and times of administration. The regulation of tissue healing and regeneration is a complex process, and even small differences in the amount of growth factors administered can lead to the opposite results. As Weibrich [15] reported, the PLT concentration required for a positive PRP effect on bone regeneration spanned a limited range. An advantageous biological effect seemed to occur when PRP with a PLT concentration of approximately 1^6^/microl was used. At lower concentrations, the effect was suboptimal, whereas higher concentrations had a paradoxically inhibitory effect. Moreover, as in this case, the combination of PRP with other treatments further complicate the interpretation of the results. The reasons for the contradictory findings about the influence of PRP on the regenerative potential of scaffolds are not completely understood although some hypotheses have been advanced. Some mechanisms have been proposed to determine the success or the failure of the treatment: the presence of collagen on experimental biomaterials (both calcium phosphate and hydroxyapatite) induces in vitro the activation of PLTs and GF release [28]; the specific surface area of rough and porous materials modulates PRP absorption and causes increased degranulation and release of the GFs from the PLTs [29]; a fibrous tissue and a foreign body giant cell reaction, which was observed when PRP was added to synthetic biomaterials, might be due to the presence of TGF, which also supports fibroblast chemotaxis [30]. Regarding these hypotheses, it is possible that the implant surface and chemical composition influence the format in which GFs are delivered and the time course of delivery during the early inflammatory phases. GFs can also affect the scaffold through the stimulation of cell resorption of the implants. Moreover, the presence of areas within the subchondral compartment that positively stained for type II collagen, including cells with hypertrophic chondrocyte morphology, suggests that the scaffold-mediated regeneration of subchondral bone followed an endochondral ossification process. To this purpose, Ranly et al. observed that PDGF and PLT gel reduced heterotopic bone formation (usually through endochondral ossification) after muscle implantation of demineralised bone matrix (DBM) even in the presence of DBM with a proven osteoinductive ability [31,32]. These authors also observed that GFs stimulate osteoclast resorption of the implants. Finally, we might also argue that the high content of angiogenic growth factors in PRP can delay and disturb cartilage regeneration and maturation with time.

From our findings, we deduce that PRP does not enhance the osteochondral regeneration capability of a collagen-hydroxyapatite scaffold in this sheep model. On the contrary, by negatively interfering with the regenerative process, the bioactive factors in the PLT concentrate produced worse results than those obtained from the use of the scaffold alone. It is possible that different PRP preparation techniques, doses and application modalities produce different and better results.

## Conclusion

Current research aims to develop innovative approaches to improve chondral and osteochondral regeneration. In this study, we tested if the addition of PRP would further improve the good results previously obtained with a newly developed biphasic scaffold alone by increasing the regeneration process of the osteochondral unit. PRP seems to be an ideal vehicle to provide numerous growth factors, and it has been widely experimented on worldwide in numerous fields because of its potential to facilitate the healing process. However, our findings demonstrated that this administration modality in the sheep model may negatively interfere with tissue regeneration. Therefore, we conclude that, even if theoretically useful for many treatments, PRP has to be carefully considered before its use in humans; the risk of negative effects and the role of many variables that may influence the final outcome need to be clarified to obtain better results and optimise the potential benefits of this technique.

## Competing interests

The authors declare that they have no competing interests.

## Authors' contributions

EK Involved in conception and design, surgical treatment

GF Involved in drafting the manuscript, surgical treatment, analysis and interpretation of data

MD Involved in surgical treatment, analysis of data

MF Involved in drafting of the manuscript, interpretation of data

FS Involved in acquisition, analysis and interpretation of data

GG Involved in acquisition, analysis and interpretation of data

IM Involved in acquisition, analysis and interpretation of data

MM Involved in conception and design, final approval of the version to be published

All authors read and approved the final manuscript.

## Pre-publication history

The pre-publication history for this paper can be accessed here:

http://www.biomedcentral.com/1471-2474/11/220/prepub
